# The Effect of Confinement Angle on Self-Colliding Aluminium Laser Plasmas Using Spectrally Resolved Fast Imaging

**DOI:** 10.3390/ma13235489

**Published:** 2020-12-02

**Authors:** Lazaros Varvarezos, Stephen J. Davitt, John T. Costello, Thomas J. Kelly

**Affiliations:** 1 School of Physical Sciences and National Centre for Plasma Science and Technology, Dublin City University, 9 D09 Dublin, Ireland; stephen.davitt2@mail.dcu.ie (S.J.D.); john.costello@dcu.ie (J.T.C.); 2 Department of Computer Science and Applied Physics, Galway-Mayo Institute of Technology, Galway Campus, T91 T8NW Galway, Ireland; mossy.kelly@gmit.ie

**Keywords:** laser produced plasmas, self-colliding plasmas, time-resolved imaging

## Abstract

In this work we investigate the effect of the confinement angle on self-colliding aluminium laser produced plasmas. More specifically, we apply V-shaped channel targets of different angles (90°, 60° and 30°) and report both broadband and filtered time-resolved fast imaging measurements on the formation of such plasmas in ambient air. Based on the broadband measurements we suggest that the plasmas formed on the two inner walls of the V-shaped channel expand normally to the surface, interact with each other and possibly stagnate. The spectrally filtered fast imaging reveals the presence of a spatial distribution of different species within the plasmas and signatures of forced recombination.

## 1. Introduction

Laser produced plasmas (LPPs) have always been pertinent to fundamental plasma research, however the rapid advance of their technological applications has placed them under a bright spotlight. Their applicability thus extends over a wide range of research fields including the extreme-UV lithography (EUVL) [1], the pulsed laser deposition (PLD) [2,3], ion acceleration [4], laboratory scale astrophysics [5,6] and fusion research [7,8,9].

In the case of colliding plasmas, two extreme scenarios may occur, namely interpenetration or stagnation [10]. The so-called collisionality parameter ζ introduced by Rambo and Denavit, allows for a distinction between interpenetration and stagnation. This parameter expresses the ratio of the separation between the two colliding plasmas to the ion–ion mean free path. Hence, when the collisionality parameter is less than unity (ζ < 1) interpenetration will prevail, whereas when the collisionality parameter is greater than unity (ζ > 1), stagnation becomes the dominant scenario.

The collisionality of the two plumes can be affected by modifying the target geometry [11,12,13,14]. Hence, different target configurations such as collinear [15,16,17,18,19,20], crossed [21] and orthogonal [22,23] geometries have been applied to investigate the collision process. Notably, all the aforementioned studies were performed in vacuo. On the other hand, a rather limited number of works involving colliding plasmas in air [24,25] have been reported in the literature.

Under ambient pressure conditions, air breakdown is possible, thus restricting the laser fluences that can be used during the experiment in order to ensure that the plasma is formed from the target material and not the background gas (for example [26]). Furthermore, laser produced plasmas, which expand in air, undergo spatial confinement, due to the interaction with the surrounding environment [27]. The spatial confinement has been examined as a potential method for improving the detection sensitivity of laser induced break down spectroscopy (LIBS). Various geometries such as cylindrical cavities [28,29] and flat obstacle(s) [30,31,32] have been implemented in order to ensure spatial confinement of laser produced plasmas in air.

Spatial confinement of laser plasmas has been shown to be a useful, versatile technique to increase signal enhancement for laser induced breakdown spectroscopy [33], ion flux enhancement [34] and increased conversion efficiencies in extreme ultraviolet lithography [35] and thus the study and understanding of confined plasmas, and their optimization using target geometry is potentially of interest.

In this work, we report broadband and filtered time-resolved fast imaging measurements on colliding plasma plumes, created on aluminium V-channel targets with varying angles (90°, 60° and 30°) and a flat target for comparison. The measurements were carried out at an ambient pressure (i.e., 1 atm) and the comparatively small angles of 30° and 60° were chosen in order to increase the relative collisional velocity, aiming to overcome the increased confinement of the plasma. Thus, the effect of confinement angle on self-colliding plasmas created in air, is examined here for the first time to the best of our knowledge.

## 2. Experimental Apparatus

**Figure 1:**
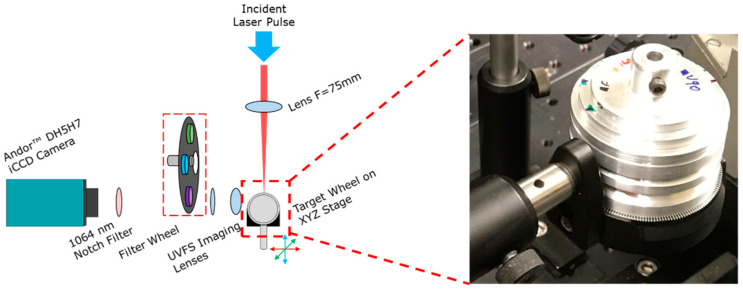
A schematic of the fast-imaging experimental setup along with a photograph of the V-channel target geometry. A plano-convex lens (F = 75 mm) was used to focus the laser beam onto the target. A pair of UV fused silica (UVFS) lenses with focal lengths 50 mm and 150 mm were applied in order to achieve a magnification 3×. Narrow bandpass filters were introduced via the filter wheel for the spectrally resolved measurements.

A schematic of the experimental setup is presented in Figure 1. Laser pulses of 18 ns (FWHM) duration at a central wavelength of 1064 nm were delivered by a Spectron^TM^ SL803 Nd:YAG laser system (Azusa, CA, USA). The pulse energy was 20 mJ focused down to a spot size of approximately 200 μm in diameter. The laser system operated at a repetition rate of 1 Hz. Based on these values a peak intensity of 0.8 × 10^9^ W/cm^2^ can be calculated.

The time-resolved images presented in this work were recorded by means of the Andor^TM^ DH5H7 Intensified Charged Coupled Device (ICCD) model camera (350–1000 nm, Belfast, UK) with 512 pixels × 512 pixels. The temporal width of the gate was set at 10 ns and the ICCD camera was synchronised to the laser pulse and could be delayed with a Stanford instruments model DG535 delay generator. Scattered laser light was rejected by a 1064 nm notch filter and neutral density filters (1%, 10% and 30%) were used to avoid saturation of the camera. For the spectrally resolved fast imaging experiments, narrow bandpass filters, exhibiting a bandwidth of less than 10 nm, were introduced in order to isolate the plasma emission due to specific transitions of the neutral atom or ions. Hence, the spatial evolution of different excited state atoms/ions could be monitored over time.

## 3. Results and Discussion

### 3.1. Broadband Time-Resolved Fast Imaging

#### 3.1.1. Flat Target

**Figure 2:**
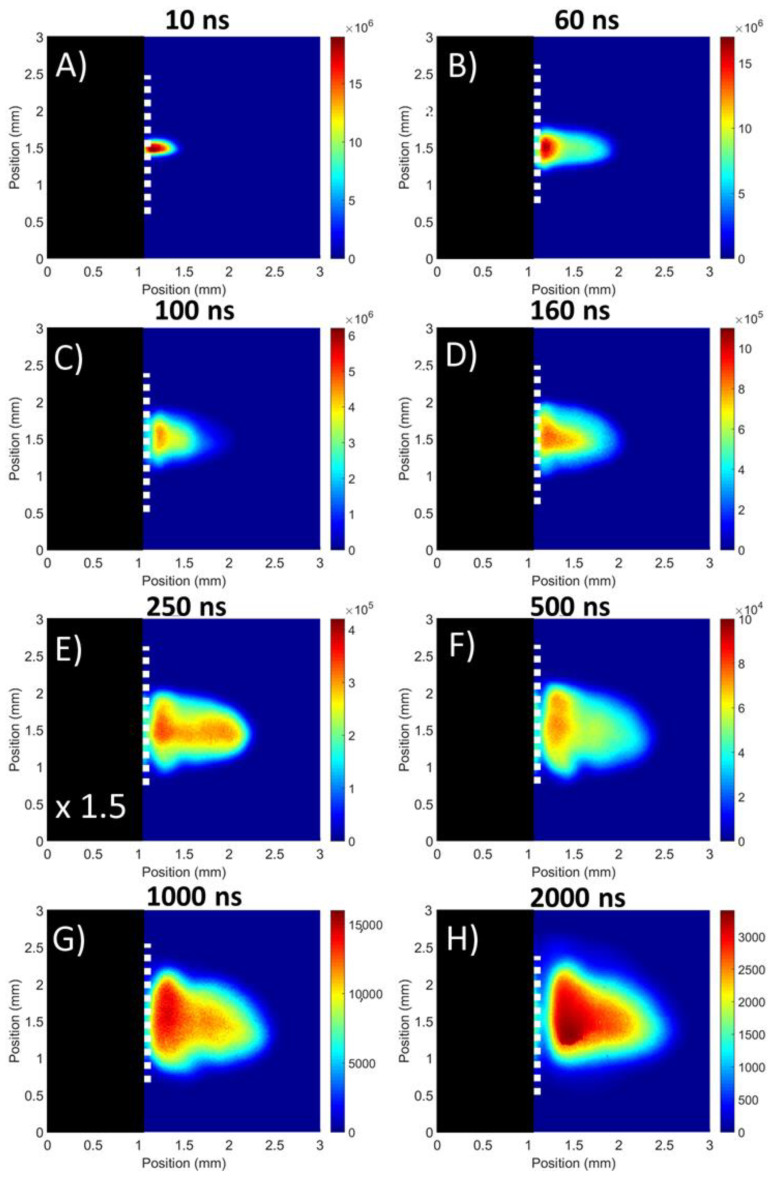
Images (**A**–**H**) of the evolution of a laser plasma created on the flat target. The broadband images have been normalised at each time-step across all targets for ease of comparison.

**Figure 3:**
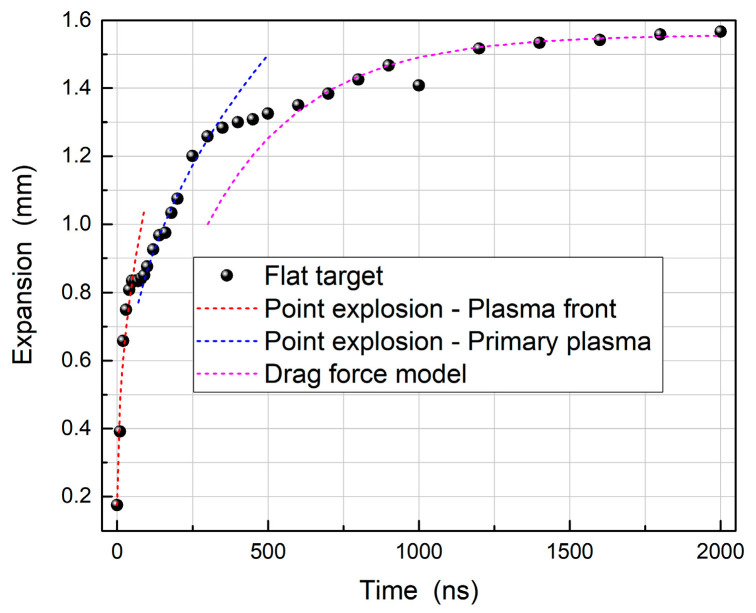
The luminous plume expansion of a laser plasma in air from a flat target. Point explosion models were fitted to the plasma front (red curve) and primary plasma (blue curve) for the early delay times while the drag force model (magenta curve) was fitted at late time delays.

In Figure 2A a set of images of the evolution of plasmas formed on a flat target is presented. In addition, the luminous plume front position graph is shown in Figure 3. The latter data were obtained from horizontal lineouts taken from the centre of the broadband images along an axis normal to the target centre point (or vertex for grooved targets). The expansion length is defined as the distance from the target at which the emission intensity of the plasma was reduced to 5% of the peak emission intensity recorded for the initial plasma position on the sensor at time t = 0 ns.

In that case, the findings suggest the presence of two components: a fast “plasma front” and a slow “primary plasma” similar to the previous observations by Wu and coworkers [36]. The presence of two components can be attributed to the laser supported detonation (LSD) wave. Specifically on the other hand, the plasma front, which is coupled to the shock wave, expands rapidly outwards due to the extra energy absorbed. Conversely, the primary plasma was left to slowly expand resulting in the observed plume splitting.

According to Figure 3 the plasma is seen to expand rapidly over the first 40 ns and this can be attributed to the faster plasma front expanding towards the laser pulse. Upon termination of the laser pulse, the plasma front can no longer gain energy to overcome the confinement by the surrounding air at atmospheric pressure. Thus, the plasma front expansion decelerated and began to cool down via radiation losses. In parallel, the primary plasma expanded at a slower rate behind the plasma front and began to be more noticeable at around 60 ns. A flattening of the luminous plume front position curve around 80 ns is noticeable from Figure 3, indicating that emission from the plasma front was decaying and, as a result, the bright primary plasma becomes the leading edge of the luminous expansion. This switching of the leading edge occurred due to the primary plasma becoming very distinct from the plasma front as can be seen from the image corresponding to the time delay of 100 ns. At a time delay of 160 ns, a deceleration of the primary plasma plume is observed. This is caused by interactions with the plasma front material, along with confinement by the atmospheric pressure air surrounding it. Furthermore, considerable lateral expansion has occurred, and the plasma has assumed a cone shape, characteristic for a laser plasma expanding in air. A sharp interface between the plasma plume and the background can be observed at a time delay of 250 ns, due to strong confinement as the plasma expands into the air at ambient pressure. The plasma then follows a slow diffusion into the surrounding air, as can be observed at 500 ns.

At later time delays, the plasma cools and the more highly charged ions have recombined with electrons to form a plume dominated by neutral and singly charged species. Eventually at 2000 ns, the plasma reached a “stopping distance” of 1.56 mm in agreement with reference [36] where similar experimental parameters were present. The dependence of the stopping distance on laser parameters such as the pulse energy and wavelength has been studied in reference [37].

#### 3.1.2. V-Channel Targets

**Figure 4:**
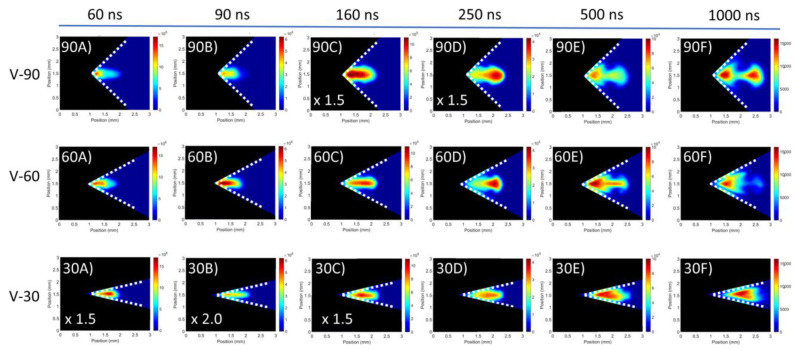
Time-resolved images of the evolution of a laser plasma created on the 90° V-channel target (**top row**), the 60° V-channel target (**middle row**) and the 30° V-channel target (**bottom row**). Image intensities are normalised with multiplicative factors shown on the bottom left where required.

**Figure 5:**
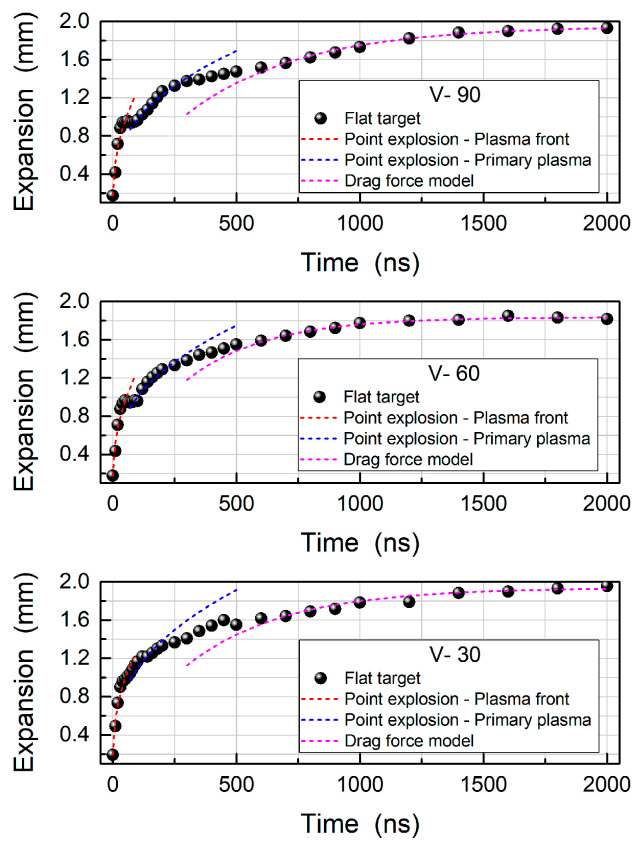
The luminous plume expansion of a laser plasma in air from the 90° V-channel target (**top row**), the 60° V-channel target (**middle row**) and the 30° V-channel target (**bottom row**). Point explosion models were fitted to the plasma front (red curve) and primary plasma (blue curve) for the early delay times while the drag force model (magenta curve) was fitted at late time delays.

Moving on to the V-channel targets, Figure 4 includes a set of images of the plasma evolution for each V-channel target. Additionally, Figure 5 shows the luminous plume front position data for the 30°, 60° and 90° target respectively. From the data presented in Figure 5 similarities between the 60° and 90° V-channel targets and the flat target are observed at early times. Specifically, the formation of an LSD wave accounted for the plume splitting due to the rapid expansion of the plasma front while the slower moving primary plasma component follows. This plasma front expansion terminated shortly after the end of the laser pulse within 40 ns. At a delay time of 60 ns, two distinct components can be observed, while at 80 ns a dip is present in Figure 5 for both 60° and 90° V-channel targets. This feature was also observed for the flat target, and it was attributed to the primary plasma becoming the leading edge of the luminous expansion. In the images corresponding to a time delay of 100 ns it can be seen that the plasma front emission decayed considerably such that it became barely visible as the primary plasma emission comes to dominate the image.

At later time delays (t > 100 ns) noticeable differences between the V-channel targets and the flat target can be observed, with the former exhibiting greater directionality as a result of the greater confinement and hence, lower lateral expansion. Such directionality is indicative of collisions that occur within the plasma. More to the point, plasmas expanding from each wall of the V-channel targets will collide at a collision plane or surface. Typically, this would occur in the vicinity of the centre of the V-channel, and the opposing lateral velocity components will cancel. This results in stagnated plasma formation and a net outward growth of the plasma away from the target, with little or no lateral component of expansion and hence greater directionality in the plasma plume expansion.

At a time delay of 160 ns, it can be observed from Figure 4 that a cylindrical shape corresponds to the V-channel plasmas as opposed to the cone shape observed for the flat target. Confinement by the background air was also evident due to the sharp plasma air interface both in the lateral and outward directions. In the case of 60° and 90° V-channel targets, such confinement created a build-up at the leading edge of the plasma, which was evidenced by the formation of a lobe-like plasma plume component at the leading edge of the plasma (see Figure 4). At 250 ns, the primary plasma was seen to split into two distinct plasma components, consisting of the lobe-shaped plasma region at the leading edge of the plume, which is now distinct from the other component. At even further time delays, the plasma component within the target vertex remained somewhat stationary, showing little or no expansion. On the contrary, the lobe-shaped plasma plume component expands away from the target. It is proposed that this lobe-shaped plasma component can be explained as follows: the component of the plasma plume located near the vertex of the target creates a pressure gradient, due to the high density, which exerts a force on the plasma region further from the target. The region coalesces into a lobe, which then moves away from the target due to the applied pressure, leading to the appearance of the two plasma components, at long time delays: a stationary plasma, close to the target vertex and a moving plasma lobe. The coalescence of plasma in ambient air into multiple components is a well-known observation going back to the days of the earliest fast photographs of laser plasmas [38]. Further confinement of the stationary plasma close to the target is attributed to shockwave reflection from the walls of the cavity, as per previous observations [39,40].

At even longer time delays, when the stationary plasma and plasma lobe have separated, the electron density and degree of ionisation in both components will be low and so that the Debye length will be long. Hence, Coulombic forces are not expected to affect the separation between the two components.

Turning our attention to the 30° V-channel target, it can be seen from Figure 4 that no dip is present in the early plasma expansion. Importantly, unlike the other two V-channel targets, it does not seem to exhibit any indication of the appearance of a separate plasma lobe. In addition, as time proceeds, expansion of the plasma plume away from the target continued to slow, and signs of lateral expansion became evident, with the plasma expanding to fill the target vertex region at a time delay of 250 ns. Between 250 and 500 ns, the plume evolved into stationary plasma, similar to the case of the other two V-channels. Furthermore, the component of the plasma plume protruding into the ambient air exhibited a flat and well-defined front edge. This stationary plasma did not expand further and exhibited uniform emission while the plasma cooled.

Assuming a two-component expansion for early times, a single fit to the full expansion data range would not, of course, be satisfactory. Instead, two separate fits of the point explosion model to the luminous front data were required, as can be seen in Figure 3 and Figure 5, one for the plasma front (red curve) and one for the primary plasma (blue curve). Thus, for early times the point explosion model was used to fit the plasma front and primary plasma. The formula for the point explosion model is given as [41]:

R(t) = α × t^n^ (1)

where R denotes the shock front position at a time t after plasma ignition. The parameters “α” and “n” are extracted from the fits and are tabulated in Table 1. More specifically, “n” describes the shape of the shock front and values of 0.4, 0.5 and 0.667, respectively describe spherical, cylindrical or planar shapes. It is clear from the extracted n values that all targets tend towards a cylindrical expansion, in line with the observation that the plasma fronts have a fast, outward expansion, along the incident laser direction, with little evidence of lateral expansion. The “α” values, which are dependent on the energy of the point explosion, indicate that all the V-channel targets show a modest, but distinct increase in this energy as the vertex angle decreases. In addition, the velocities were calculated for the early time delays (0–60 ns) resulting in an increase of 19%, 18% and 24% for the 90°, 60° and 30° V-channel target respectively, due to the increase of the point explosion energy.

Concerning the primary plasma that became dominant after the initial rapid expansion of the plasma front, we tabulated the extracted values also in Table 1. In that case, we obtained n values of 0.4, for all three vertex angles, meaning that the expansion was quite spherical. Similar to the plasma front case, the V-channel targets give rise to an increased point explosion energy compared to the flat target. This increase in the primary plasma expansion rate supports the proposition that the 30° V-channel target does not show a dip in the luminous expansion data since the primary plasma and the plasma front have expanded at similar rates. As a matter of fact, the two point explosion fits corresponding to the primary plasma (blue curve) and plasma front (red curve) expansions in the 30° V-channel case, overlap each other smoothly. In contrast, for the other targets the two curves were distinct and exhibited a short flat region in the luminous expansion plots as the most distant luminous front swaps from the plasma front to the primary plasma.

Remarkably, at a time delay of 160 ns, an increased degree of lateral confinement is observed as the target geometry became tighter. However, as the plasma remained confined, even when not in contact with target walls, it was proposed that this is not just due to geometric confinement but also arises from the plasmas formed on the target walls. As a result we could state that the tighter the target angle the higher the confinement. When the plasma material arrives at the collision plane the faster lateral expansion components in the 30° and 60° V-channel targets formed well-defined regions or “harder” stagnation, while the slower lateral components from the 90° V-channel formed a less-defined “softer” stagnation.

At longer delay times (t > 600 ns), the fitting of the luminous plasma front position data was performed using the drag force model (green curve). In that case, the equation used to fit the data is given as [42]:

R(t) = R_0_ × [1 − e^−βt^] (2)

where R_0_ is the stopping distance of the plasma plume and β is the slowing coefficient such that R_0_ × β = υ_0_ with υ_0_ being the initial velocity. The extracted parameters are tabulated in Table 2. A first point to make, is that the extracted β values, for the 30° and 90° V-channel targets are smaller, thus resulting in longer stopping distances of 1.95 mm and 1.94 mm respectively. On the other hand, the 60° V-channel target exhibits a somewhat smaller stopping distance of 1.84 mm due to the unexpectedly smaller slowing parameter. This observation may be attributed to the fact that these measurements are based on the luminous plasma front position. The emission from the plasma column in the 60° V-channel target was seen to decay away at late time delays and as such this would have a negative going impact on the luminous plasma front position.

By 1000 ns the plasmas have all but stopped expanding and peak intensities are all approximately equal in all cases. However it can be seen that each of the V-channel targets exhibited strikingly different behaviours. The 90° V-channel target had two bright regions of intense emission, the extended plasma lobe and the stationary plasma located near the V-channel vertex. The 60° V-channel target had intense emission from the stationary plasma while in contrast to the 90° V-channel case, the plasma column displayed much weaker emission. The 30° V-channel did not show any indication of a distinct separate lobe formed at any time delay and the entire plasma appeared to behave as one stationary plasma. It is worth pointing out that the stationary plasmas within the V-channels at this point all exhibited similarities in emission intensities and distribution.

At time delays beyond 1000 ns, the intensities of the images for the different targets began to decay at different rates. The 30° and 60° V-channel target intensities fell away at the fastest rates, while the 90° V-channel target was closer to the flat target case. This decrease in emission was proposed to be due to confinement of the targets. More specifically, it was seen that the V-channel targets had an early increase in emitted radiation and this was attributed to confinement increasing the recombination rates, with the rate scaling inversely with the vertex angle leading to a concomitant increase in radiative losses. It is proposed that the increased collisional rates for the 60° and 30° V-channel target plasmas means that the radiative losses occurred more quickly for these cases than the less well confined flat and 90° V-channel cases. This is supported by previous literature studies where time-resolved shadowgraphy was used in tandem with fast imaging (e.g., [39,40]). In these papers, which have similar experimental conditions to those present in our work, the authors observe spectral enhancement and persistence in the confined plasma case which scales with the level of confinement. In the case of [40], they find that as the confinement increases, so too does the spectral enhancement. Thus, this agrees with our observations.

Turning to the individual stationary and lobe plasmas at rather long time delays, when they have been separated for some time, it is observed that the plasma lobe emission intensity decayed more quickly than the emission intensity for the stationary plasma, particularly for the 60° V-channel target case, where the plasma column was not visible in the corresponding image at 2000 ns. Importantly, the stationary plasmas formed at what would be the location of the collision plane from the initial plasmas expanding from the target walls. They exhibited many of the characteristics one would expect from a stagnation layer, for example, they were stationary showing little to no expansion and they had quite uniform intensity distributions, which decayed more slowly than regions further from the target vertex, e.g., in the lobe plasma region.

### 3.2. Filtered Time-Resolved Imaging

In addition to time-resolved broadband fast imaging, filters corresponding to transitions of several ion stages of Al (394.4 nm, 396.2 nm), Al^+^ (466.3 nm) and Al^2+^ (569.6 nm) were used to perform spectrally resolved fast imaging measurements, aiming to track the evolution of each ion stage within the plasma.

#### 3.2.1. Flat Target

**Figure 6:**
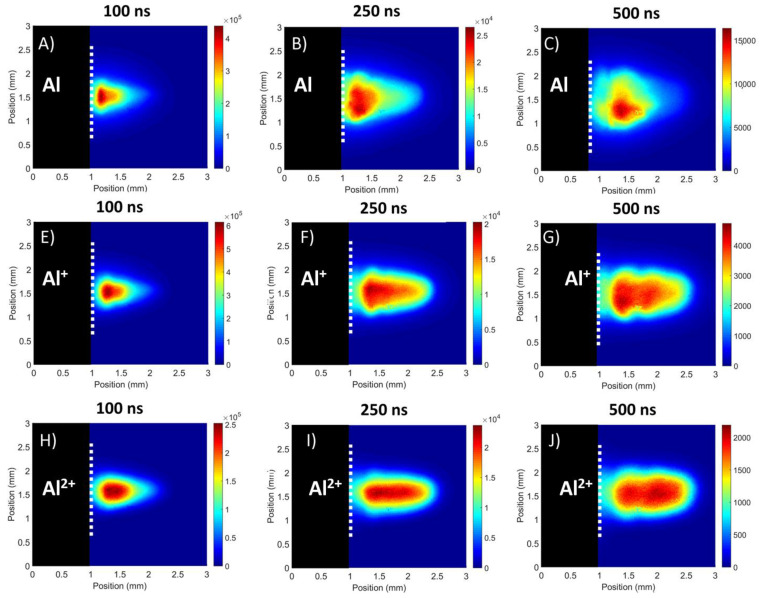
Spectrally filtered images (**A**–**J**) of the flat target showing the different distributions of the Al, Al^+^ and Al^2+^ species for three time delays.

Figure 6 shows spectrally resolved images for a set of time delays (100, 250 and 500 ns) when the flat target was used. One can observe that, for early times, the differences in the intensity distributions between the neutral and ionized species were insignificant. At these early stages, the plasma emission was dominated by continuum radiation, thus no spectral signature of any specific charge state was expected to be present. However, at later time delays (i.e., 250 ns) line emission becomes more important resulting in images that exhibit noticeable differences in intensity distributions, for different charge states. This can be explained as follows: electrons tend to move away from the target faster than ions. Thus, an ambipolar field is formed, which exerts an attractive force on the charged ions, accelerating them in a forward direction, resulting in an ion stage distribution that depends on the charge state. The slower neutral Al atoms tend to stay close to the target while the ions will move away more rapidly with increasing charge state.

At a time delay of 500 ns, besides the variation in the spatial distribution of the different species present, a similar variation is observed in the relative emission intensity. Thus, for atomic aluminium (Al) a drop of 40% in the total image intensity between time delays of 250 and 500 ns was recorded, whereas the Al^+^ and Al^2+^ images exhibited overall drops of 75% and 90% respectively. This is attributed to the charged species undergoing recombination as the plasma expands and cools resulting in a reduction in the number density of more highly charged ions and an increase in neutral atoms. Since the recombination rate depends on the ion stage, one expects that drop in image intensity to be fastest for the highest charge states.

#### 3.2.2. V-Channel Targets

**Figure 7:**
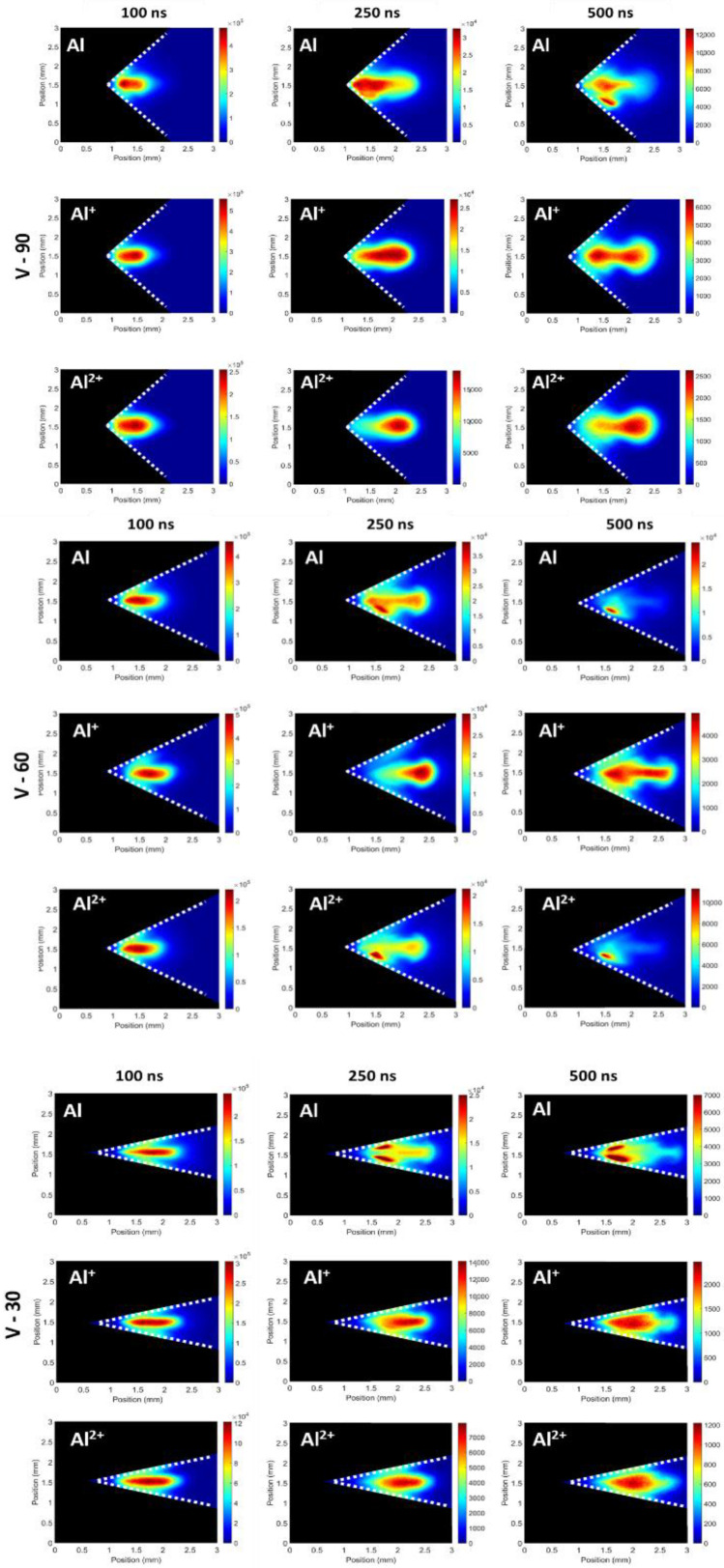
Spectrally filtered images of the 90°, 60° and 30° V-channel targets showing the different distributions of the Al, Al^+^ and Al^2+^ species at different time delays.

Similar images were recorded for the V-channel targets, presented in Figure 7. For a time delay of 100 ns, similar behaviour to the flat target is observed as a result of the dominance of the continuum emission. At longer time delays, the continuum emission decayed away allowing for evidence of a spatial distribution to appear. Examining the images associated with the 90° V-channel target at 250 ns, it is observed that the Al^2+^ emission took place in the plasma lobe at the leading edge of the plasma. On the contrary, the neutral emission was mainly located close to the target vertex in the vicinity of the stationary plasma, while the Al + emission bridged the two regions. At a time delay of 500 ns, the plasma lobe appeared to cool faster than the stationary plasma and the strong emission from Al^+^ and Al^2+^ ions decayed to levels comparable with the stationary plasma, resulting in a flattening of the intensity distribution across the plasma plume similar to that observed for the flat target at the same time delay.

In the case of 60° and 30° V-channel targets at 250 ns, emission from localized spots was found to exceed that from the main plasma where the neutral aluminium is concerned. At 500 ns, emission from such spots becomes the dominant contribution. It is proposed that these bright spots are due to the phenomenon of “forced recombination” [43,44]. Specifically, enhanced emission is observed when plasmas collide with “cold” electron donor surfaces increasing recombination rates. Thus, it is suggested that at the plasma–target interface there might be an increase in the population of excited neutral Al species as Al^+^ ions recombine with electrons donated from the target wall. The neutral Al emission did not exhibit such bright spots for the 90° V-channel target, possibly due to the looser confinement of the plasma in this geometry. Evidence of forced recombination was also observed in the case of Al^2+^ emission when the 60° V-channel target was applied.

The Al^+^ emission did not display evidence of forced recombination as no bright spots can be observed in neither the 60° nor 30° V-channel targets. Hence, it resembled the flat and 60° V-channel targets, with intense emission at the front of the plasma at a time delay of 250 ns, showing strong localisation of Al^+^ in that region of the image before it evolved into a more uniform distribution across the plasma. In a similar manner, this is attributed to the plasma column cooling faster than the stationary plasma.

## 4. Conclusions

To conclude with, both broadband and filtered time-resolved fast imaging measurements were reported in order to obtain insight into the expansion dynamics of plasmas formed on the various flat and V-channel targets (90°, 60° and 30°). The findings from the broadband measurements at early stages, suggest a two-component expansion: a rapidly expanding plasma front and the slower primary plasma. At a time delay of = ≈60 ns the primary plasma took over as the plasma front decayed. The plume expansion data were fitted using two different plasma expansion models. Upon decreasing target angle, the point explosion model yielded higher energy, as a result of the increasing initial velocity. At longer time delays the expansion was described by the drag force model, with the V-channel targets showing greater final stopping distances than the flat target.

Around 160 ns, the 90° and 60° V-channel targets were seen to form two distinct components, the stationary plasma and the plasma lobe. The stationary plasma was present at the location where one would expect the stagnation layer arising from the collision of two plasmas formed on the inner walls of the target to form. Furthermore, it was found to exhibit many of the characteristics, expected from a stagnation layer. However, further investigation and plasma diagnostics would be required in order to confirm the role of such stationary plasmas as proxies for stagnation layers.

Results from the spectrally filtered measurements showed the presence of a spatial distribution of the various species within the plasmas, with the Al^2+^ species moving towards the leading edge of the plasma while the neutral Al species tended to stay close to the target surface in each case. The Al^+^ ions were seen to bridge these regions with a good distribution over the length of the plasma while showing a slight preference towards the leading edge. Some evidence for forced recombination was obtained, with the plasma from the V-channel targets showing spots of intense Al emission due to interactions with the target walls.

Our results broadly agree with previous spatial confinement studies and indeed we saw the same features in broadband and spectrally filtered imaging. Specifically, we saw emission persistence increasing with confinement for atomic and ionic species. This is attributed to forced recombination at the walls of the channels. We also observed plume splitting and coalescence, which again is in general agreement with previous studies of this kind. Therefore, we could conclude that the V-shaped channels were relatively simple geometries that can result in the same kinds of signal enhancement observed in other spatial confinement geometries. Thus, they are potentially a useful target shape for LIBS (where signal enhancement is important) or EUVL (where conversion efficiency is important).

## Figures and Tables

**Table 1:** Fitting parameters obtained by fits to the point explosion model for the plasma front and the primary plasma. * indicates that the value was forced to this value by the boundaries set in the fitting.

**Plasma Front**	**Flat**	**V90°**	**V60°**	**V30°**
α	0.11	0.12	0.12	0.13
n	0.46	0.48	0.48	0.46
**Primary Plasma**	**Flat**	**V90°**	**V60°**	**V30°**
α	0.11	0.13	0.13	0.14
n	0.41	0.40	0.40*	0.40 *

**Table 2:** Parameters obtained by fits to the drag force model for time delays longer than 600 ns.

	Flat	V90°	V60°	V30°
R_0_	1.56	1.95	1.84	1.94
β	3.00 × 10^−3^	2.20 × 10^−3^	3.10 × 10^−3^	2.50 × 10^−3^
